# The Microbial Communities in Male First Catch Urine Are Highly Similar to Those in Paired Urethral Swab Specimens

**DOI:** 10.1371/journal.pone.0019709

**Published:** 2011-05-13

**Authors:** Qunfeng Dong, David E. Nelson, Evelyn Toh, Lixia Diao, Xiang Gao, J. Dennis Fortenberry, Barbara Van Der Pol

**Affiliations:** 1 Department of Biology, University of North Texas, Denton, Texas, United States of America; 2 Department of Computer Science and Engineering, University of North Texas, Denton, Texas, United States of America; 3 Department of Biology, Indiana University, Bloomington, Indiana, United States of America; 4 Department of Bioinformatics and Computational Biology, M.D. Anderson Cancer Center, University of Texas, Houston, Texas, United States of America; 5 Section of Adolescent Medicine, Department of Pediatrics, Indiana University School of Medicine, Indianapolis, Indiana, United States of America; 6 Indiana University School of Public Health, Bloomington, Indiana, United States of America; 7 Indiana University School of Medicine, Indianapolis, Indiana, United States of America; Johns Hopkins University Bloomberg School of Public Health, United States of America

## Abstract

Urine is the CDC-recommended specimen for STI testing. It was unknown if the bacterial communities (microbiomes) in urine reflected those in the distal male urethra. We compared microbiomes of 32 paired urine and urethral swab specimens obtained from adult men attending an STD clinic, by 16S rRNA PCR and deep pyrosequencing. Microbiomes of urine and swabs were remarkably similar, regardless of STI status of the subjects. Thus, urine can be used to characterize urethral microbiomes when swabs are undesirable, such as in population-based studies of the urethral microbiome or where multiple sampling of participants is required.

## Introduction

Urethral swabs robustly sample urethral pathogens and have historically been specimens of choice for STI research in men. However, swab collection causes considerable discomfort and has been identified as a disincentive for routine STI screening. Urine is now the CDC-recommended sample type for nucleic acid based diagnostics [1]. Data supporting this recommendation strongly suggest that the organisms colonizing the urethral epithelium, including intracellular pathogens, are present in urine in sufficient quantities to be diagnostically relevant.

We recently characterized microbiomes of first-catch urine specimens from adult men using 16S rRNA allele sequencing [2]. Results of our study, and of cultivation- dependent studies performed in the past, showed that first-catch urine from adult men can contain complex microbiomes, and that the composition of these microbiomes may be relevant to STI and urogenital tract disease [2], [3], [4], [5], [6]. Despite the utility of urine specimens for diagnostic purposes, it is unclear whether this specimen type will be equally useful for studies of the male urethral microbiome. Compared to urines, urethral swabs, for example, could more efficiently sample organisms that tightly adhere to epithelial cells or those which reside in biofilms. Urine may also contain microorganisms from other segments of the urinary system including the bladder and prostate. If it could be shown that urine and urethral swab specimens broadly and similarly sample urethral bacteria, this would increase feasibility of population-based or longitudinal studies using urine samples to characterize urethral microbiomes.

In this study, we collected paired urine and swab specimens from 32 men who visited an urban STD Clinic in Marion County, Indiana, and compared their microbiomes using multiplex 16S rRNA PCR and deep pyrosequencing. Our results show that the microbiomes in male first-catch urine and urethral swab specimens are nearly identical, independent of STI or urethral inflammation status.

## Methods

### Subjects

Participants were recruited from the Bell Flower clinic, an urban STD clinic in Indianapolis, IN. Specimens from 32 men, 18 years or older (median 28 y/o) were evaluated. All subjects provided written informed consent and The Indiana University-Clarian Institutional Review Board (IRB) approved all procedures for patient specimen collection and data handling. This IRB which serves all patient-related facilities on campus including the Bell Flower Clinic at which all participants were recruited.

### Specimens

Dacron-tipped swabs were inserted approximately 1–3 cm into the urethra and rotated for 3–5 seconds. The swabs were immediately placed in vials containing 2.0 ml of phosphate buffered transport medium and were stored at −80°C within 18 hours of collection. Subjects provided urine immediately following swab collection. Urine was stored without additives at −80°C.

*STI and inflammation testing*: Urine was tested for *Chlamydia trachomatis* and *Neisseria gonorrhoeae* using a commercial diagnostic test (Amplicor CT/NG PCR; Roche Diagnostics, Indianapolis IN). *Trichomonas vaginalis* was identified using a modification of the Amplicor assay that included primers and probes specific to *T. vaginalis* DNA [7]. Urethritis was assessed by microscopic counting of polymorphonuclear leukocytes (PMN) per high power field (HPF); patients with counts of ≥5 PMN/HPF were considered positive for urethritis.

### DNA isolation

Urethral swab samples were thawed and vigorously vortexed for 1 min. 1 ml of the resulting suspensions, or 5 ml of thawed urine, was pelleted by centrifugation for 15 min at 4,000×*g* at 4°C. DNA was harvested from the cell pellets using a Qiagen DNeasy (Qiagen Inc., Valencia CA) tissue extraction kit. Genomic DNA was eluted in nuclease-free water and stored at 4°C until16S rRNA PCR and sequencing. Mock specimens were processed in parallel with patient specimens to monitor for reagent contamination.

### Multiplex 16S rRNA PCR and pyrosequencing

V1-V3 region 16S rRNA PCRs included 2 µl of urine gDNA preparation, Phusion high fidelity DNA polymerase (New England Biolabs, Ipswich, MA) and oligonucleotide primers 27F, which additionally contained an adaptor sequence B, and 534R coupled to the A adaptor sequence and a unique barcode (454 Life Sciences, Branford CT). The forward primer (A-534R) sequence was 5′-ccatctcatccctgcgtgtctccgactNNNNNNNNATTACCGCGGCTGCTG-3′ where the sequence of the A adaptor is shown in lowercase letters, and N represents a unique barcode specific to the primer. The reverse primer (B-27F) sequence was 5′-cctatcccctgtgtgccttggcagtctcagagaGTTTGATCCTGGCTCAG, where the B adaptor sequence is shown in lowercase letters. PCR Amplicons were purified by Qiaquick gel extraction kit (Qiagen) and quantified by Quant-It HS double stranded DNA assay (Invitrogen, Carlsbad CA). Emulsion PCR and 454 library generation steps were performed according to the manufacturer's instructions (454 Life Sciences). Sequencing was performed on a Roche/454 GS-FLX Titanium system at the Indiana University Center for Genomics and Bioinformatics, Bloomington IN.

### Bioinformatics and statistical analysis

Sequences were sorted to specimens only if they perfectly matched primer barcode sequence. Sequences that did not contain perfect matches to primer barcodes, were less than 200 bp in length, and or had average quality scores of less than 25 were discarded. The primer and barcode sequences were then trimmed from the remaining sequences. All of the sequences were BLASTed [8] against the human genome and sequences with significant similarity (E-value threshold 10^−10^) were excluded from subsequent analyses. Taxonomic classifications were assigned using RDP Classifier v.2.2 [9]. Four different RDP classifier confidence cutoffs were applied to the same data set for most analyses: 90%, 80%, 70%, and 60%. Hierarchical clustering was performed using Spearman's rank correlation coefficient as a measure of distance. Paired t-test, Wilcoxon sum rank test, McNemar's test, and Kolmogorov-Simirnov tests were performed to compare microbial distributions after normalizing sequence read counts for each assigned taxon to total high-quality sequence reads in the specimen.

## Results

### Urine and urethral swabs yield similar proportions of high quality and classifiable 16S rRNA sequences

33,629 and 30,419 high quality 16S rRNA sequences were identified in urine and swab specimens, respectively. On average, 84.0% (SD±17.3%) of the urine and 87.7% (SD±17.84%) of the swab sequences could be classified to the genus level by RDP II classifier with ≥90% confidence. Similar results were observed using relaxed RDP classifier confidence cutoffs (i.e., 80%, 70%, and 60%; data not shown). These results indicate that urine and swab specimens yielded similar proportions of high quality 16S rRNA sequences, and that neither specimen type differentially sampled unclassified taxa.

### Bacterial genera and the proportions of these genera are similar in urine and urethral swab specimens

At a 90% RDP confidence cutoff, an average of 18.47 (SD±6.83) and 14.44 (SD±9.19) bacterial genera were identified within individual urine and swab specimens, respectively. All analyses below resulted in similar findings when relaxed RDP classifier confidence cutoffs were applied (i.e., 80%, 70%, and 60%, data not shown). McNemar's χ^2^ test was used to examine if the genera were present in the same proportions in urine and swab specimens, separately for groups of men who did (n = 10) and did not have STI (n = 22).

All 88 bacterial genera identified in urine specimens from the STI positive men were also present in the swab specimens. McNemar's χ^2^ tests indicated only proportions of *Propionibacterium spp.* differed among urine (0.87%) and swabs (0.39%) (p = 0.04). The only significant difference in distribution of the 131 genera identified in the STI negative men was *Corynebacterium* (p<0.001, FDR = 0.2%) which was enriched in urine compared to swab specimens (11.9% vs.7.6%, respectively). McNemar's tests indicated four genera were also enriched in urine prior to adjustment for multiple sampling in this group: *Pelomonas* (p = 0.003, FDR = 16.70%), *Propionibacterium* (p = 0.004, FDR = 19.18%), *Staphylococcus* (p = 0.023, FDR = 60.69%), and *Finegoldia* (p = 0.023,FDR = 60.69%). *Propionibacterium*, *Staphylococcus* and *Corynebacterium spp.* are all abundant components of superficial skin flora. Thus, this result could mean that there is a slight bias towards sampling of this flora by urine.

**Figure 1:**
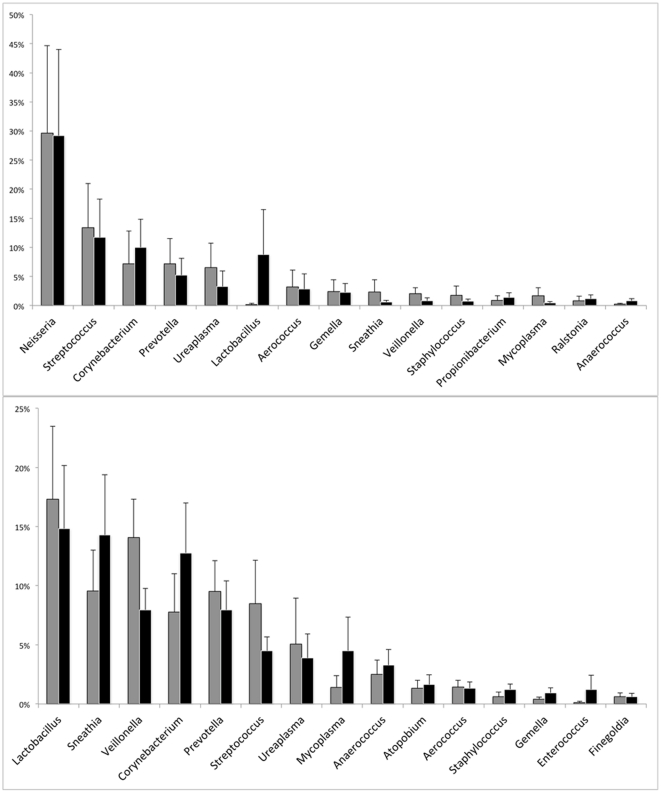
The proportions of bacterial genera in urine and swab specimens are similar. The y-axis shows percentage classified sequence reads corresponding to each genera, bars corresponding to urine and swab are in gray and black, respectively. Error bars indicate one unit of standard error. The 15 most abundant genera, corresponding to 89.48% and 83.52% of classifiable sequences from STI positive and negative specimens, respectively, are shown. (A). STI (positive test for *C. trachomatis*, *N. gonorrhoeae*, and or *T. vaginalis*) positive group (n = 10), (B) STI negative group (n = 22).

We also used paired t-tests to compare the relative abundance of sequences in the two specimen types, again in groups corresponding to STI status (fig. 1). In the STI positive group, the relative abundance of 98.9% (87/88) of the genera did not differ; this test also indicated that the proportion of *Propionibacterium spp.* sequences was elevated in urine specimens (p = 0.02, FDR = 15.9%). In the STI negative group, the abundance of 98.5% (i.e.,129/131) genera did not differ by t-test. *Dialister spp.* were significantly enriched in urines (p = 0.04, FDR = 11.9%) but were not relatively abundant in either urine or swab specimens (0.50% and 0.22%, respectively). *Veillonella spp.* sequences were significantly enriched in swab specimens (p = 0.02, FDR = 11.90%) and were relatively abundant components of both urine and swab microbiomes (7.96% and 8.49% in urine and swabs, respectively). This suggests that swabs sampled *Veillonella spp.* slightly more efficiently than did urine.

### Distributions of bacteria in individual urine-swab pairs are highly similar

**Figure 2:**
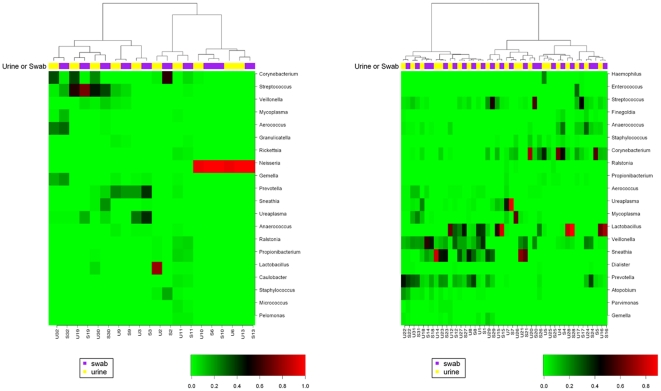
Hierarchical clustering sorts specimens into urine swab pairs. Spearman's rank correlation coefficients were calculated using relative abundance of the 20 most abundant genera in each specimen, which account for 90.91% and 86.39% of classifiable sequences in swabs and urines, respectively. Yellow indicates urine, purple indicates swab specimen. The color gradient indicates relative abundance of the genera in each specimen (red for most abundant bacteria). (A). STI positive group (n = 10). (B) STI negative group (n = 22). Note that most swab and urine specimens from individual subjects cluster together.

Because our above analyses considered urine and swab specimens in groups, we also used Kolmogorov-Smirnov (KS) testing to assess the similarity of microbiomes in individual (corresponding) urine swab pairs. Bacterial distributions did not significantly differ in any of the 10 STI positive sample pairs. Of the 22 specimen pairs from STI negative men, only two differed by KS test (subject#15, p = 0.0497, FDR = 54.68%; and subject #26, p = 0.0246, FDR = 54.19%). Separately, hierarchical clustering of all urine and swab specimens was performed using pair-wise Spearman's ranked correlation coefficients. Supporting KS test results, the closest match of most individual urine or swab specimens was usually the corresponding specimen (Figure 2). Interestingly, clustering analysis indicated that the two specimens from subject 15 were more similar to each other than to all other specimens in the group of healthy men (Figure 2). Collectively, these results suggested that first-catch urine and urethral swabs similarly sampled male urethral microbiomes.

## Discussion

The utility of first-catch urine specimens versus urethral swab specimens for microbiome analysis had not been compared previously, and there is an important advantage of collecting specimens of the former type. Here we show that the microbiomes of first-catch urine and urethral swab pairs are highly similar. The findings are consistent with studies that that have shown that these specimens perform similarly with specific nucleic acid based STI diagnostic tests.

Caveats include that urine can sample microbial communities from the upper urogenital tract and bladder. Incidences of renal, bladder, and prostatic diseases increase with age. Thus, it is possible that urine specimens from older men will be less representative of the distal urethra. The significance of this is unclear because there is little data regarding culture-independent characterization of microorganisms in the prostate, bladder and or kidney. We believe caution is warranted in extending results of this study to such men and or other populations with increased risk of UTI. Another limitation was that the population in our study was recruited from an STI clinic, so these results cannot necessarily be extended to populations of healthy men.

We previously reported differences in the urine microbiomes of groups of men with and without symptoms of STI [2]. In this study, the swab and urine defined microbiomes of sub-groups of men who did and did not have symptoms of urethritis were similar (data not shown). Thus, we now have the option of using urine specimens rather than urethral swabs to answer microbiome-related questions regardless of the presence of STI or urethritis.

Self-administered and clinician-collected vaginal specimens can similarly measure vaginal microbiomes [10]. This observation has facilitated studies where the goal is to understand the relationship between composition of female urogenital tract microbiomes and STI risk. Similar longitudinal studies have not been reported in men. However, two recent studies of male urine and the coronal sulcus showed that most of the abundant bacterial genera in these microbiomes are associated with the vaginal flora of healthy women and women with bacterial vaginosis [2], [11]. We and other groups have speculated that this implies broader exchange of urogenital microbiomes may result from vaginal sexual exposures than is previously appreciated [2], [11]. Vaginal *Lactobacillus spp.* protects against some STI [12], [13] whereas the opposite is true of BV associated microorganisms, such as *Prevotella spp.* [14]. Our observation that both lactobacilli and various BV associated genera are abundant in microbiomes of some men might mean that these bacteria are also pertinent to STI epidemiology in men. Testing this will require longitudinal surveys of male urogenital microbiomes in the contexts of incident sexual exposures and STI. We believe our results show that urine is appropriate, and is a useful but minimally invasive specimen for such studies.
